# Immune regulatory mechanisms of different exercise methods promoting Parkinson’s rehabilitation: A narrative review

**DOI:** 10.1097/MD.0000000000044035

**Published:** 2025-08-15

**Authors:** Wen Ma, Xiaotong Yuan, Youhan Liu, Qinglu Wang, Yaohua Zhang, Panpan Dong, Caixia Zhou

**Affiliations:** a Graduate School of Education, Shandong Sport University, Jinan, Shandong, China; b Key Laboratory of Biomedical Engineering & Technology of Shandong High School, Qilu Medical University, Zibo, China.

**Keywords:** exercise rehabilitation, inflammatory factors, Parkinson disease, running exercise, Tai Chi

## Abstract

Parkinson disease (PD) is the second largest and most common neurodegenerative disease globally, following Alzheimer disease. Its pathological features include the deformation and loss of dopaminergic neurons in the substantia nigra pars compacta of the midbrain, as well as the aggregation of α-synuclein in the form of Lewy bodies. This leads to motor symptoms such as resting tremors, muscle rigidity, bradykinesia, and postural instability, as well as non-motor symptoms including cognitive, emotional, and sleep disorders. Currently, PD is mainly treated by medication and surgery. Medication, though widely used, has limited efficacy and causes adverse reactions. With the intensification of global aging and the annual increase in the incidence of PD, the limitations of existing treatment approaches have become increasingly prominent, and there is an urgent need to explore safer and more effective treatment strategies. Numerous clinical studies have demonstrated that exercise rehabilitation training can not only effectively ameliorate the motor and non-motor symptoms of PD patients, but also promote the generation of neurotrophic factors, neurotransmitters, and hormones, and regulate the dopaminergic system. Therefore, an in-depth exploration of the mechanisms and effects of different exercise rehabilitation training methods in the treatment of PD holds great significance for refining the comprehensive treatment plan for PD and enhancing the quality of life of patients. This article will conduct a comprehensive review of the mechanisms and effects of various exercise rehabilitation training methods in treating PD.

## 1. Introduction

Parkinson disease (PD), also known as “paralysis agitans,” is a common neurodegenerative disease that primarily affects the middle-aged and elderly population. Its core pathological features are the progressive degeneration and loss of dopaminergic neurons in the substantia nigra of the midbrain, leading to a significant decrease in dopamine levels in the brain. At the same time, Lewy bodies appear in the cytoplasm of the remaining neurons.^[1–3]^ When the dopamine levels in the striatum decrease by more than 70% to 80%, patients gradually develop typical motor symptoms, such as resting tremors, bradykinesia, muscle rigidity, and postural balance disorders. Along with the progression of the disease, non-motor symptoms such as anxiety, depression, paresthesia, and cognitive decline also become more prominent.^[4]^

Existing studies have shown that the occurrence of PD is closely related to multiple factors such as genetics, environment, and aging. Under the combined effect of genetic and environmental factors, pathological changes such as abnormal aggregation of α-synuclein (α-syn), mitochondrial dysfunction, oxidative stress injury, lysosomal system dysfunction, and activation of immune inflammatory response occur in the preclinical stage of the disease. These pathological processes are intertwined, accelerating the degeneration of dopaminergic neurons and promoting the progression of the disease.

With the intensification of global aging, the incidence of PD has been increasing year by year. The long-term care needs caused by the disease have imposed a heavy economic burden on patients’ families and society.^[5,6]^ However, the current treatment methods for PD mainly rely on drug replacement therapy. Although it can relieve symptoms temporarily, it cannot block the progression of the disease, and there are problems such as the attenuation of therapeutic effects and frequent adverse reactions. Therefore, it is of urgent practical significance to deeply explore the pathophysiological mechanisms of PD and find effective intervention strategies to improve the prognosis of patients and reduce the social medical burden. As a non-pharmacological intervention, exercise rehabilitation has been confirmed to play a positive role in improving motor function and quality of life in patients with PD. However, previous studies mostly took it as a single intervention measure, focusing on the macro-effect evaluation of clinical symptom improvement, and lacking in-depth exploration of the regulatory mechanisms of pathological processes at the molecular and cellular levels.^[7,8]^ This article will focus on the regulatory effects of exercise rehabilitation training on the pathophysiological process of PD and its potential mechanisms for a review.

## 2. Review methodology

In the present narrative review, ethical approval was waived as the study involved the analysis of publicly available data and did not include human participants, thus eliminating the need for institutional review board approval or informed consent. This narrative review was conducted to synthesize and critically evaluate existing literature on the immune regulatory mechanisms of different exercise methods promoting Parkinson rehabilitation, with a particular focus on their roles in modulating immune cell function, regulating cytokine profiles, and reducing neuroinflammation. The review methodology adhered to a systematic approach to ensure comprehensive coverage of relevant studies while maintaining clarity and precision in the synthesis of findings.

Literature inclusion criteria: this study selects Chinese and English literatures published within the past 10 years, covering clinical research, basic experiments, and review articles. The research subjects should involve PD patients or animal models and focus on exercise rehabilitation training. The intervention measures include aerobic exercise, dance therapy, Tai Chi, and resistance training, etc, and the training frequency, intensity, and duration must be clearly specified. The content of the literature should report on the impact of exercise rehabilitation training on the motor and non-motor symptoms, neurophysiological indicators, or quality of life of patients.

## 3. Current status of PD treatment

Currently, the main treatments for PD include medication and surgical interventions.^[9]^ Traditional medication treatments encompass dopamine replacement therapy, anticholinergic agents, amantadine, and dopamine receptor agonists. The mechanisms of these anti-PD drugs are complex. In the striatum, levodopa, dopamine receptor agonists, and monoamine oxidase B inhibitors all exhibit dopaminergic effects, while anticholinergic drugs and amantadine act on postsynaptic receptors of other neurotransmitters. Amantadine affects N-methyl-D-aspartate glutamate receptors and has dopamine-releasing effects, potentially providing neuroprotection. Although these drugs can alleviate symptoms to some extent, they cannot halt the progression of PD and long-term use often leads to various complications.^[10]^ Surgical treatments include thalamotomy or pallidotomy, neural transplantation, and deep brain stimulation. However, the long-term effects of surgical treatments remain uncertain, the costs are high, and they cannot reverse the degeneration of dopaminergic neurons. Therefore, PD treatment primarily focuses on symptomatic relief without effectively improving patients’ motor functions. In recent years, clinical studies have adopted various types of exercise rehabilitation training, such as balance training, aerobic exercise, gait training, and progressive resistance training. Research indicates that exercise rehabilitation interventions can be as effective as medication in treating PD, significantly enhancing patients’ walking and balance abilities, and positively impacting functional recovery. Consequently, exercise rehabilitation training, as an effective non-pharmacological intervention, has garnered increasing attention. However, the mechanisms through which different exercise training methods affect PD remain unclear.

**Figure 1.**
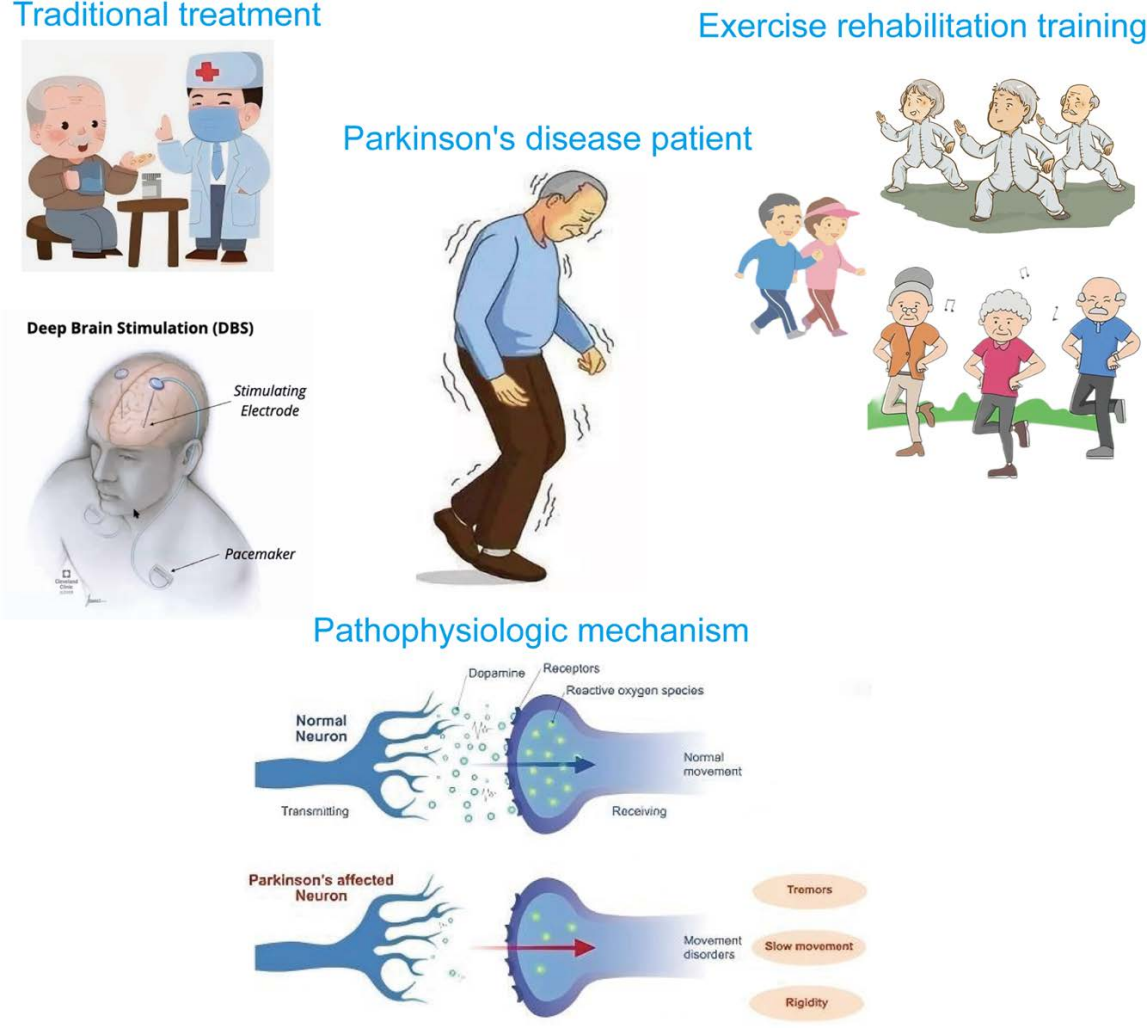
The pathological mechanisms and treatment methods of Parkinson disease The figure mainly illustrate the pathological mechanisms of Parkinson disease, traditional drug and DBS surgical treatments, and exercise rehabilitation training, including running training, Tai Chi training, and dance training. DBS = deep brain stimulation.

This review summarizes the effects and mechanisms of various exercise rehabilitation training methods in PD patients, aiming to provide optimal exercise rehabilitation strategies for PD patients to slow disease progression and improve quality of life (Fig. 1).

## 4. Current status of exercise rehabilitation training for PD treatment

Exercise rehabilitation is a form of therapy that utilizes movement to improve physical function and enhance motor abilities in patients. Based on the theory of motor learning, exercise rehabilitation focuses on analyzing and addressing abnormal or missing components that contribute to motor dysfunction in order to design targeted exercises that promote brain function and restore normal motor skills.^[11]^ In a comparative study conducted by Jesús Seco Calvo et al^[12]^ 27 PD patients were divided into 2 groups: 1 receiving medication treatment alone and the other receiving a combination of medication and exercise rehabilitation. After 8 months of rehabilitation exercises, both groups showed significant reductions in the Unified PD Rating Scale scores, but the combined medication and exercise rehabilitation group demonstrated more pronounced improvements. Margaret K Y Mak et al^[13]^ conducted rehabilitation training using methods such as treadmill training, brisk walking, Nordic walking, dancing, and Tai Chi in PD patients. The results showed short-term or long-term beneficial effects of these interventions, with Tai Chi exercise showing sustained benefits for up to 6 months and slowing down the progression of PD. Elizabeth Maria Atterbury et al^[14]^ highlighted gait and posture abnormalities as major motor symptoms that need to be addressed in PD patients. They randomly assigned 40 idiopathic PD patients into 2 groups: TG (exercise rehabilitation training group) and BG (traditional rehabilitation training group). The study demonstrated significant improvements in gait after exercise rehabilitation training in the TG group, suggesting the positive impact of exercise rehabilitation on gait and posture abnormalities in PD patients. Extensive research conducted by G. Earhart et al^[15]^ has shown that exercise rehabilitation training is as effective as medication treatment in the management of PD. They have identified various exercise methods that positively contribute to motor function recovery in PD patients, emphasizing the importance of exercise rehabilitation training for patients, their families, and healthcare professionals. In conclusion, exercise rehabilitation training has shown promising results in improving motor symptoms and functional abilities in PD patients. It has become an important therapeutic approach in the management of PD, providing significant benefits to patients and contributing to their overall well-being.

## 5. Effects of different exercise rehabilitation training methods on PD

Exercise rehabilitation training has been shown to improve functional abilities, gait, balance, and quality of life in PD patients. Evidence from animal studies on neurodegenerative diseases suggests that exercise training can enhance the production of neurotrophic factors, neurotransmitters, and hormones, promoting processes such as synaptic plasticity, neurogenesis, angiogenesis, and autophagy.^[16]^ Previous animal studies have indicated that exercise may benefit PD patients by inhibiting oxidative stress, repairing mitochondrial damage, and promoting the production of growth factors.^[17]^ Exercise training has beneficial effects on the degeneration of the central nervous system by modulating glutamatergic and dopaminergic systems. Additionally, the beneficial effects of exercise therapy are associated with the regulation of autophagy, apoptosis, inflammation, α-syn aggregation, and mitochondrial function. Exercise may reduce the reactivity and inflammation of microglia by regulating various metabolic and transcription processes.^[18]^ Glycogen synthase kinase-3 (GSK-3) is a major regulatory factor for the balance of pro-inflammatory and anti-inflammatory mediators in immune cells, including microglia.^[19]^ GSK-3 stimulation activates the release of pro-inflammatory cytokines such as interleukin-1Beta (IL-1β), interleukin-6, and tumor necrosis factor α, while inhibiting the release of anti-inflammatory cytokines such as interleukin-10.^[20]^ Exercise may activate some known extracellular signals that inhibit GSK-3, including brain-derived neurotrophic factor (BDNF).^[21,22]^ It is also possible that long-term exercise increases the synthesis of neurotrophic factors without promoting inflammatory proliferation and activation of astrocytes. This is because dopamine neurons protected by neurotrophins do not degenerate and therefore do not generate signals for mobilizing pro-inflammatory responses. In a mouse model of PD, the potential benefits of exercise can be explained by increased expression of anti-inflammatory cytokines, decreased levels of pro-inflammatory cytokines, and reduced activation of microglia.^[23]^ Furthermore, the benefits of exercise may be attributed to the reduction of mitochondrial dysfunction caused by decreased expression of α-syn.^[24]^ Furthermore, the positive effects of exercise may be mediated by enhancing antioxidant defense against neurotoxins and reducing glutamate-driven excessive activity, thereby reducing the loss of dopaminergic cells.^[25]^ Different exercise rehabilitation training methods have varying effects on the progression of PD. Therefore, it is crucial to investigate the mechanisms by which the most effective exercise training methods for symptom relief in PD patients exert their effects.

### 5.1. Aerobic running training

Aerobic exercise training is exercise performed under sufficient oxygen supply, mainly targeting rhythmic, moderate, or low intensity, and long duration strength exercises of major muscle groups. The energy for these activities comes from the aerobic metabolism of carbohydrates and fats in the body. Aerobic exercise training is considered the best choice for improving lifelong health. A related study on PD mice found that aerobic exercise is associated with regulating dopamine and glutamate neurotransmission, altering synaptic plasticity, and increasing cerebral blood flow.^[25]^ Aerobic exercise training has neurorecovery and neuroprotective effects, possibly achieved by regulating BDNF to support neuronal synaptic formation and vascular generation, inhibiting oxidative stress reactions, and improving mitochondrial function.^[26]^ Aerobic exercise training can increase BDNF expression levels, induce transcription factors, and regulate gene expression related to neuronal proliferation, survival, and inflammatory responses, thereby inhibiting neurodegenerative changes. Exercise on a treadmill can increase the levels of BDNF and glial-derived neurotrophic factor in the striatum of rats with PD model.^[27]^ It has been reported that 4 weeks of treadmill training can improve motor impairment in a PD rat model by regulating mglur2/3-mediated glutamatergic transmission.^[28]^ In addition, long-term treadmill training can reduce the loss and transmission of dopamine cells in PD animal models, and reduce protein oxidation caused by neurotoxins.^[29,30]^ Eight weeks of treadmill training increased the dopamine D2 receptor binding potential in PD patients, indicating that the therapeutic effect of exercise is related to the neuroplasticity of the dopaminergic pathway.^[31]^ Treadmill exercise can inhibit the formation of Lewy bodies in the substantia nigra striatum of PD rat models, protecting dopaminergic neurons and fibers in the substantia nigra striatum and exerting a therapeutic effect.^[32]^ Additionally, treadmill exercise can increase Nrf2 and γGCLC levels in PD animal models, reverse HO-1 upregulation, Nrf2/γGCLC downregulation, and dopaminergic neurodegeneration in the substantia nigra striatum.^[33]^ Treadmill training inhibits reactive astrocyte and microglia activation and rotenone-induced cerebellar apoptosis in PD rats.^[34]^ Regular aerobic running exercise therapy for neurodegenerative diseases is adaptive due to its ability to activate miR-3557/324 and regulate the downstream Ca^2+^/calmodulin-dependent protein kinase 9 (CaMKs) signaling pathway.^[35]^ CaMKs coordinate gene expression in the mTOR pathway, regulate uch-l1 levels, and contribute to improving the pathogenesis of PD rat models or alleviating neurodegenerative changes.^[35]^ Aerobic running exercise involves the movement and cognitive processing networks, which are beneficial for their plasticity reshaping and may lead to skill recovery in complex syndromes such as PD.^[36]^ Aerobic running exercise has also been shown to be a booster for adult neurogenesis, increasing the number of newborn cells and promoting their integration into established circuits.^[37]^ It has been reported that after moderate aerobic activity, there is an upregulation of systemic anti-inflammatory mediators and regulatory T cells, a balance in the activity of the myeloid cell lineage including the balance of resident microglia, and improved function of the antioxidant system and associated repair proteins.^[38]^ In summary, aerobic running exercise is an effective method for treating PD patients. As a mainstream approach to functional recovery therapy, it enhances the plasticity of movement-related structures and improves motor learning ability, positively impacting the movement, cognition, emotions, and quality of life of PD animal models and patients.

### 5.2. Tai Chi training

Tai Chi is a traditional Chinese martial art that utilizes continuous, bending, and spiraling body movements to control breathing.^[39]^ It can improve aerobic capacity, muscle strength, balance, and motor control, as well as reduce stress and anxiety in the elderly.^[40]^ Tai Chi has been widely studied for various medical conditions, including PD. A review of Tai Chi for general health and fitness reported excellent evidence of improving balance and endurance.^[41]^ In recent years, there has been an increasing amount of research on the molecular mechanisms by which Tai Chi training benefits PD. These studies suggest that peripheral inflammation and peripheral immune involvement play a role in the pathogenesis and progression of PD. Potential mechanisms may include enhancing brain network function, reducing inflammation, improving amino acid metabolism, energy metabolism, and neurotransmitter metabolism, and reducing susceptibility to dopamine degeneration. Disruptions in PD metabolites and metabolic pathways are primarily related to amino acid metabolism (such as glycolic acid, L-focal, and arginine biosynthesis), energy metabolism (such as L-malic acid, 3-phosphoglyceric acid, urea cycle, tricarboxylic acid cycle (TCA) cycle, and beta-oxidation of very-long-chain fatty acids), and neurotransmitter metabolism (such as adenosine).^[42]^ L-arginine is involved in nitric oxide synthesis and plays a key role in the pathogenesis of PD by affecting oxidative stress and energy metabolism.^[43]^ TCA cycle enzyme deficiency and mitochondrial dysfunction, which regulate neuroinflammation and neurodegeneration, have also been observed in PD.^[44]^ Adenosine, coupled with its specific receptors, acts as an upstream neuromodulator of neurotransmitters such as acetylcholine, glutamate, gamma-aminobutyric acid, and dopamine, and participates in the regulation of various bodily functions.^[45]^ In a clinical study,^[46]^ it was found that Tai Chi training improved amino acid metabolism, energy metabolism, and neurotransmitter metabolism in PD patients. After Tai Chi training, pro-inflammatory cytokines were downregulated, and plasma cytokines IL-1β, interleukin-5, interleukin-7, interleukin-9, interleukin-13, monocyte chemoattractant protein-1, macrophage inflammatory protein-1alpha were relatively downregulated, while granulocyte-macrophage colony-stimulating factor was upregulated. The downregulation of IL-1β was positively correlated with improved BBS scores. The improvement in Unified PD Rating Scale was related to enhanced default mode network function, reduced L-malic acid and 3-phosphoglyceric acid levels, and increased adenosine and Huntingtin interaction protein 2 (HIP2) mRNA levels. Additionally, improvements in arginine biosynthesis, urea cycle, TCA cycle, and beta-oxidation of very-long-chain fatty acids were also observed through Tai Chi training.^[46]^

HIP2 is an E2 ubiquitin-conjugating enzyme that participates in protein degradation through the ubiquitin–proteasome system pathway.^[47]^ Impairment of the ubiquitin–proteasome system system is associated with protein aggregation, leading to inflammation and oxidative stress.^[48]^ In a PD model, decreased expression of HIP2 results in impaired spontaneous motor function and increased susceptibility to dopaminergic degeneration.^[49]^ The expression of HIP2 mRNA was downregulated in 20 PD patients, and after 1 year of Tai Chi training, the expression of HIP2 mRNA was upregulated, accompanied by improved motor function.^[49]^ In a larger PD cohort, Tai Chi training was found to reverse the downregulation of HIP2 mRNA, and this change was correlated with improved motor function in PD patients after Tai Chi training, suggesting that Tai Chi training may reduce susceptibility to dopaminergic degeneration in PD patients.^[46]^ In this study,^[50]^ a total of 136 differentially expressed genes were found in PD patients after Tai Chi training, mainly involved in neutrophil activation, T cell activation, NOD-like receptor, and interleukin-17 signaling pathways. The upregulation of RNASE7 expression in blood leukocytes of PD patients after Tai Chi exercise suggests that Tai Chi may be involved in the regulation of immune abnormalities in PD patients. Therefore, Tai Chi training can be considered as a beneficial exercise rehabilitation therapy for treating PD patients. In the future, larger clinical cohort studies can be conducted to further explore the molecular mechanisms of Tai Chi training in the pathogenesis of PD, aiming to improve functional impairments in PD patients and provide new targets for future research.

### 5.3. Dance training

Dance training is a rhythmic exercise that stimulates multiple levels of the nervous system as a sensory motor activity, including movement planning and execution, sensory integration, and cognitive processing. Dance training may affect multiple functional domains in PD. Some case studies, quasi-randomized pilot trials, and systematic reviews suggest that dance has beneficial effects on both motor and non-motor symptoms of PD, including balance, gait, quality of life, mood, attention, and memory.^[51–56]^ Dance is a typical multitask exercise involving aerobic capacity, balance and posture control, gait, as well as cognitive skills related to music and rhythm cues,^[57–59]^ which meet the requirements of PD patients’ physical therapy clinical guidelines. Dance intervention is associated with increased activation of the prefrontal cortex and enhanced functional connectivity between the basal ganglia, cerebellum, and prefrontal cortex.^[60]^ Studies have shown that dancing can produce positive motor and non-motor outcomes and improve the quality of life in healthy older adults and individuals with mild to moderate PD.^[61,62]^ Dance requires matching movement patterns with the timing and beat of music. Music helps activate areas such as the striatum and release biochemical mediators such as endorphins^[63]^ and dopamine.^[64]^ Zhang et al^[65]^ also proposed a possible mechanism, mentioning that rhythmic stimulation in dance interventions for PD patients is an external cue that can increase striatal activity, promote movement, and compensate for the lack of dopaminergic stimulation. Salim poor et al^[66]^ also suggested that the musical rhythm in dance can promote dopamine release in the basal ganglia of PD patients. Combining exercise, relaxation, and social interaction with music can further reduce stress and anxiety, thus reducing neuroinflammation and oxidative stress.^[67–70]^ A published case study by Batson et al^[71]^ demonstrated a significant increase in network connectivity between the basal ganglia and the premotor cortex after dance intervention, suggesting possible mechanisms of neuroplasticity and motor changes induced by dance in PD patients, providing valuable evidence for prospective research on dance intervention. Another study^[72]^ found that after 6 months of dancing, the gray matter volume of the left middle frontal gyrus (associated with voluntary motor control) significantly increased, and BDNF levels significantly increased, while the fitness group did not show significant changes. Over a total of 18 months, dance increased the volume of the Para hippocampus (related to working memory and episodic memory retrieval), although BDNF levels almost returned to baseline. However, in the fitness group, brain volume and BDNF levels remained stable during the 18-month training period. In conclusion, the potential role of dance training in PD is worth further exploration. Research on the mechanisms of dance training’s impact on PD and better regulation of dance training movements to improve PD symptoms is needed.

### 5.4. Other types of exercise

In recent years, various types of exercise therapy have been reported to have significant therapeutic effects on both the motor and non-motor symptoms of PD patients. In addition to the 3 types of exercises mentioned in this review, other forms of exercise training such as aquatic exercise, robot-assisted gait training, progressive resistance training, exergames, qigong, and yoga have also played a significant role in improving symptoms in PD patients.^[7,73]^ Aquatic exercise takes advantage of the buoyancy, viscosity, hydrostatic and hydrodynamic forces of water to provide a unique mechanical advantage. When combined with the warmth of water, it is associated with reduced pain and stiffness. The viscosity of water provides a special source of natural resistance, which can promote different training tasks by providing adaptive resistance for muscle strengthening. These characteristics of the aquatic environment allow individuals with postural instability, high fall risk, leg weakness, and gait disturbances to successfully exercise when it is not feasible or safe on land.^[74,75]^ According to relevant studies, water-based exercise is safe and can improve motor performance, quality of life, and balance in PD patients. Robot-assisted gait therapy can improve PD patients’ bradykinesia, motivation, freezing, rigidity, gait, leg flexibility, and posture. Compared to treadmill training, robot-assisted gait therapy helps improve average speed, stride length, and step width in PD patients.^[76]^ Progressive resistance training (PRT) is a form of exercise training that involves performing a small number of repetitions until fatigue, allowing sufficient rest between repetitions to recover, and increasing additional resistance as the ability to generate muscle strength increases.^[77]^ It has been reported that PRT has positive effects on muscle strength, motor function, and endurance in PD patients.^[78,79]^ In another study, PRT showed improvements in cardiovascular autonomic dysfunction in PD patients.^[80]^ In conclusion, PRT has beneficial effects on improving motor symptoms, sleep dysfunction, and quality of life in PD patients, particularly in terms of muscle strength. Exergames are electronic games that can be operated through body movements, providing visual, tactile, and auditory stimulation to enhance multi-sensory interaction, and improve gait function in PD patients.^[81]^ Qigong is a traditional Chinese exercise that combines techniques of meditation, mind-body regulation, and breathing exercises.^[82]^ Qigong has positive effects on muscle stiffness, balance, and hand–eye coordination, making it an effective rehabilitation treatment for PD patients.^[83]^ A systematic review and meta-analysis have shown that practicing qigong can improve postural control, balance, and reduce the risk of falls in PD patients.^[84]^ Yoga is a practice that integrates breathing exercises, postures, and mindfulness meditation. Yoga practitioners can enhance and maintain cognitive and attentional awareness of thoughts, body, and the present moment.^[85]^ Corjena et al investigated the effects of yoga on PD patients for 12 weeks and concluded that yoga is feasible and can be used as a complementary treatment to improve motor performance.^[86]^ Therefore, yoga training can effectively improve psychological and balance disorders in PD patients. Furthermore, larger-scale clinical studies and more rigorous experiments are needed to confirm the effects of exercise rehabilitation training on PD patients and further explore the underlying mechanisms of exercise therapy to advance the field of PD rehabilitation treatment.

## 6. Conclusions and outlook

In conclusion, the role and mechanism of exercise rehabilitation training in the treatment of PD are gradually becoming a research focus. Numerous studies have shown that various exercise rehabilitation training methods, such as aerobic running, Tai Chi, and dance training, can effectively improve both the motor and non-motor symptoms of PD patients and enhance their quality of life. These training methods can not only positively regulate the production of neurotransmitters and neurotrophic factors but also regulate inflammatory factors, thereby delaying the process of neurodegeneration. For example, aerobic running can increase the expression of BDNF, promoting the formation of neuronal synapses and angiogenesis; Tai Chi can improve amino acid metabolism, energy metabolism, and neurotransmitter metabolism, reducing the inflammatory response; dance training can significantly improve the motor functions of patients, such as balance and gait. Meanwhile, the rhythm of music stimulates the release of dopamine, improving mood and cognitive functions. In addition, other forms of exercise, such as aquatic exercise training, robot-assisted gait training, progressive resistance training, exergaming, Qigong, and yoga, have also demonstrated remarkable effects in improving the symptoms of PD patients.

It is worth noting that recent studies, such as those conducted by the Al-Kuraishy team, have found that factors such as hyperhomocysteinemia, the classical and non-classical axes of the renin–angiotensin system, and abnormal prolactin levels are all closely related to the onset and progression of PD.^[87–89]^ Additionally, the NF-κB/NLRP3 inflammasome axis plays a crucial role in patients with type 2 diabetes mellitus complicated by PD.^[90]^ The revelation of these new mechanisms provides potential targets for the action of exercise rehabilitation training. Batiha et al^[91]^ found that the active components of *Commiphora myrrh* exhibit significant anti-inflammatory and immunomodulatory effects, suggesting that exercise may delay the pathological progression of PD by regulating endogenous substances and activating similar pathways. Al-Kuraishy et al^[92]^ showed in their study on sitagliptin intervention in non-diabetic COVID-19 patients that drugs can affect disease progression through unique mechanisms, implying that different exercise modalities may also have specific immunomodulatory pathways. Exercise rehabilitation training may be able to regulate the above-mentioned pathological pathways, forming a synergistic effect with other treatment methods, and further optimizing the comprehensive treatment strategy for PD.

However, current research still has many limitations, such as small sample sizes, short follow-up periods, and insufficient depth in mechanism studies. Future research should focus on conducting large-scale clinical studies to verify the long-term efficacy and action mechanisms of different exercise rehabilitation training methods. Meanwhile, follow-up studies could further explore the synergistic effects of different exercise rehabilitation training combinations, utilize multi-omics technologies to dissect the molecular regulatory networks of exercise intervention, and carry out large-sample, long-term follow-up clinical trials to validate the durable efficacy of exercise rehabilitation training. At the same time, it is necessary to explore the applicability of various exercise rehabilitation training methods at different stages of PD and develop personalized rehabilitation programs to maximize the promotion of patients’ functional recovery and quality of life improvement.

As a non-pharmacological intervention, exercise rehabilitation training has significant therapeutic effects and potential neuroprotective properties. With the continuous in-depth study of its action mechanisms, exercise rehabilitation training is expected to become an important part of PD treatment and bring more benefits to patients. A scientific and reasonable exercise rehabilitation training program can not only slow down the progression of the disease and improve both the motor and non-motor symptoms of patients but also reduce the burden on society and families. Therefore, in-depth research and promotion of the application of exercise rehabilitation training in PD treatment have important clinical and social significance.

## Author contributions

**Conceptualization:** Youhan Liu, Yaohua Zhang, Panpan Dong, Caixia Zhou.

**Formal analysis:** Xiaotong Yuan, Qinglu Wang.

**Funding acquisition:** Qinglu Wang.

**Investigation:** Qinglu Wang.

**Writing – original draft:** Wen Ma, Caixia Zhou.

**Writing – review & editing:** Yaohua Zhang, Panpan Dong, Caixia Zhou.
